# Rapid plant regeneration, analysis of genetic fidelity, and neoandrographolide content of micropropagated plants of *Andrographis alata* (Vahl) Nees

**DOI:** 10.1186/s43141-021-00122-5

**Published:** 2021-01-26

**Authors:** Sathish Shekhappa Kadapatti, Hosakatte Niranjana Murthy

**Affiliations:** Department of Botany, Karnatak University, Dharwad, 580003 India

**Keywords:** Andrographolide, *Andrographis alata*, Neoandrographolide, Medicinal plant, Micropropagation

## Abstract

**Background:**

*Andrographis alata* (Vahl) Nees is a medicinal plant which was reported to have the highest concentration of neoandrographolide that has several therapeutic values. Natural populations of *Andrographis alata* are dwindling due to destruction of natural habitat and over exploitation. Therefore, in vitro propagation of *Andrographis alata* was undertaken, and successful method is presented here.

**Results:**

Micropropagation of *Andrographis alata* was realized on MS nutrient medium augmented with BAP (10 μM), and multiple shoots were regenerated from nodal explants. Induction of roots was attained from shoots on ¼ concentration of MS nutrient medium supplemented with IBA (1 μM). Randomly amplified polymorphic DNA (RAPD) and inter-simple sequence repeat (ISSR) analysis showed that there is genetic fidelity in the regenerated plants. Reverse phase high performance liquid chromatographic analysis of regenerated plants showed the presence of neoandrographolide, equivalent to that of mother plants.

**Conclusions:**

Successful in vitro regeneration of *Andrographis alata* is presented here, and it is quite useful for its mass multiplication. The micropropagated plants are useful for restoration of plants in nature and for utilization by the pharmaceutical industry for extraction of neoandrographolide.

## Background

*Andrographis* belonging to Acanthaceae comprises of many important species which are used as medicinal plants. *Andrographis paniculata* is a well-known species which is valued in Ayurveda, Unani, Siddha, and Chinese system of medicine for curing snake bite, fever, common cold, dysentery, malaria, diabetes, and hypertension [1]. Diterpenoids, flavonoids, quinic acids, xanthones, and noriridoids are major phytochemicals extracted from *A. paniculata* [1]. Andrographolide, deoxyandrographolide, neoandrographolide, 14-deoxy-11,12-didehydroandrographolide, and isoandrographolide are the prominent diterpenoids which are responsible for antiangiogenic, antiatherosclerotic, antitumor, antidiabetic, antiinflammatory, antioxidant, immunostimulant, hepatoprotective, neuroprotective, and insecticidal activities [2–4].

*Andrographis alata* (Vahl) Nees is an herbaceous species found distributed in South India [5] which contains varied flavone glycosides including 5,7,2’,6’-oxygenated flavone glycosides and 5,2’,6’-trihydroxy-7-methylflavone-2’-*O*-ß-D-glucopyronoside [6–8]. Dalawai et al. [9] reported that *A. alata* contains 33.21 mg/g DW of neoandrographolide, and neoandrographolide was proved as a potent antioxidant, antiinflammatory, antimalarial, and hepatoprotective phytochemical [10, 11]. Recent molecular docking combined molecular dynamics studies have demonstrated that neoandrographolide is a promising candidate for SARS-CoV-2 (Covid-19) treatment [12]. *Andrographis alata* is a rare and endemic species which is distributed in South India, and natural populations are dwindling due to destruction of natural habitat and over exploitation [13]. In view of the above, the current study was undertaken to develop in vitro methodology for propagation of *A. alata*, and successful method is presented here for large scale multiplication of plants.

## Methods

### Plant material and initiation of cultures

*Andrographis alata* was collected from Nagapuri forest, Hassan district, Karnataka, India, and were maintained in Botanical garden. Identification of plant species was confirmed by Prof. S. R. Yadav, Department of Botany, Shivaji University, Kolhapur, India, and voucher specimen was maintained at Herbarium, Shivaji University, Kolhapur, India. Nodal explants were sterilized by using aqueous mercuric chloride (0.1%; w/v, Sigma, USA) for 5 min. The explants (5 mm in length) were cultured on MS nutrient medium [14] supplemented with 2.5, 5.0, 7.5, and 10.0 μM BAP, KN, 2-iP, and TDZ (HiMedia, India) individually and sucrose (3%; w/v). The cultures were maintained in culture room wherein temperature, light, and relative humidity were set at 25 ± 2 °C, 16 h light (50 μmol m^−2^ s^−1^)/8 h dark, and 60% respectively.

### Induction of roots from shoots

Regenerated shoots were individually cultured onto ½ and ¼ strength MS nutrient medium containing 3% (w/v) sugar, supplemented with 1.0, 2.0, 5.0, and 10 μM of IAA, IBA and NAA respectively (HiMedia, India) for induction of roots.

### Acclimatization of plants

Micropropagated plants (5 cm in height) were transplanted to pots containing equal concentration of soil and cocopeat, and plants were reared in growth chamber where in temperature, light, and relative humidity were set at 25 ± 2 °C, 16 h light (50 μmol m^−2^ s^−1^)/8 h dark, and 60% respectively.

### Analysis of genetic fidelity of micropropagated plants

DNA was isolated from in vitro regenerated and mother plants of *A. alata* using fresh leaves as per method of Murray and Thompson [15]. Randomly amplified polymorphic DNA (RAPD) was carried out using OPA-01, OPA-02, OPA-04, OPA-08, OPA-09, OPC-07, OPG-03, and OPG-10 primers. Similarly, inter simple sequence repeat (ISSR) analysis was carried out using UBC-811, UBC-815, UBC-818, UBC-823, UBC-827, and UBC-864 primers (Operon, USA). The polymerase chain reaction was carried out using 20 ng template DNA, Taq buffer 1.5 mM MgCl_2_, dNTPs (1 mM/μl), *Taq* polymerase (2 units), and primer (5 pm) in a reaction mixture volume of 20 μl in an Eppendorf master cycler for 45 cycles. Amplified DNA products were run on 1.0% agarose gel prepared in 0.5x TAE buffer, stained with ethidium bromide, and bands were visualized under UV light. All molecular biology chemicals were obtained from HiMedia Laboratories, Mumbai, India.

### Quantitative analysis of neoandrographolide

Quantitative estimation of neoandrographolide in the leaf and stem methanolic extract of *Andrographis alata* was carried out as per the protocol of Dalawai et al. [9] and Pholphana et al. [16] with minor modification by using high performance liquid chromatography (Shimadzu prominence unit with degasser DGU-20A 5R, low-pressure quaternary pump LC 20 AD). Chromatographic separation was carried out using Nova-Pak’s reversed phase C18 column (4 mm, 4.6 mm × 250 mm). The mobile phase consisted of 30% acetonitrile in water and 0.70 ml/min flow rate. The injection volume was 20 μl. The temperature of the column was controlled at 25 °C, and samples were detected using PD-M20A photo diode array detector. The neoandrographolide was identified by comparing retention time of samples with reliable standard chromatographic peaks at 205 nm, and neoandrographolide concentration was calculated using absorbance intensity (mAU) against standards. The data are expressed as mean ± standard deviation (SD, *n* = 3).

### Data analysis

Randomized block method was followed for establishment of experiments. There were six replicates for each treatment. The pooled data of three repeated experiments is presented in the tables. Analysis of variance treatment was applied to all the data using Duncan’s multiple range test.

## Results

### Multiple shoot regeneration

**Fig. 1:**
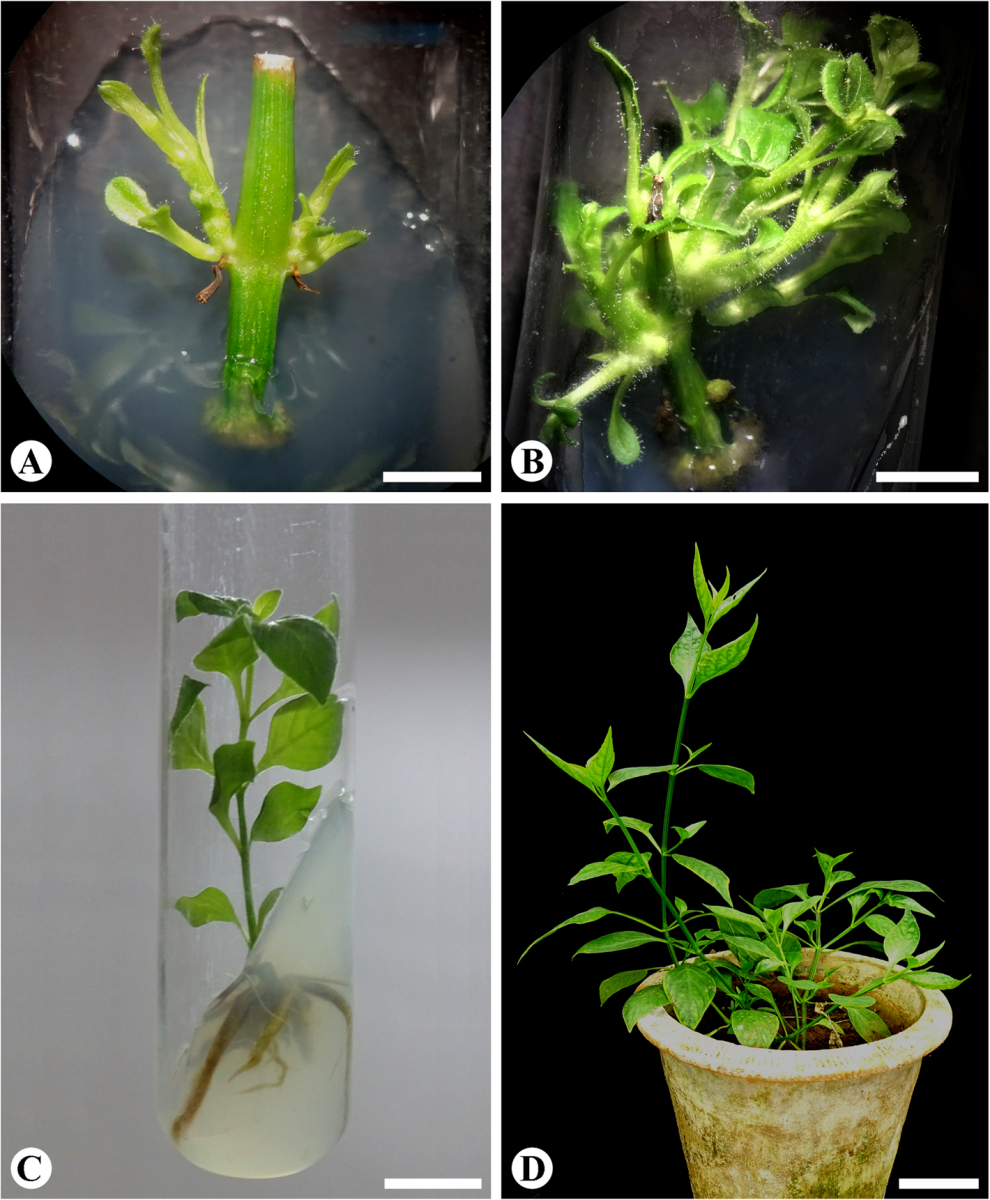
Multiple-shoot regeneration from the nodal explants *Andrographis alata*. **a** Induction of axillary shoot buds on MS nutrient medium supplemented with 10 μM BAP after 2 weeks of culture (bar = 0.25 cm). **b** Multiple shoots regenerated on MS nutrient medium supplemented with 10 μM BAP after 4 weeks of culture (bar = 0.62 cm). **c** Adventitious root induction from regenerated shoots on ¼ strength MS nutrient medium supplemented with 1 μM IBA after 4 weeks of culture (bar = 1.25 cm). **d** Four-week-old plants transferred to pots containing soil and cocopeat (bar = 7.75 cm)

Nodal explants cultured on MS medium containing several cytokinins produced multiple shoots from axillary meristem (Fig. 1a). Medium without growth regulators have not influenced shoot development from nodal explants (Table 1). Among the four cytokinins (BAP, 2-iP, KN, and TDZ) tested, BAP-supplemented medium induced highest shoot regeneration frequency (83.33%), number of shoots per explants (12.20 ± 0.64), and shoot length (1.68 ± 0.06 cm) (Table 1). Of the various concentration of BAP tested (2.5, 5.0, 7.5, and 10 μM), 10 μM BAP was superior in induction of axillary shoots, and optimum of 12 shoots were regenerated from nodal explants on this medium (Fig. 1b; Table 1). Medium containing 2-iP and KN induced 1-2 axillary shoots, whereas medium supplemented with TDZ induced only callus formation from the explants (Table 1). The shoots developed from the nodal explants were excised, and the original explants were transferred to shoot regeneration medium (MS + 10 μM BAP) and new shoot developed again from the nodal regions. The explants so transferred continued to produce shoots up to third cycle.

**Table 1:** Response of nodal explants of *Andrographis alata* cultured on MS medium supplemented with cytokinins

Growth regulator	Concentration (μM)	Percentage of regeneration	Mean number of shoots per explants	Average shoot length (cm)
Control	0.0	0	0 ± 0^f^	0 ± 0^g^
KN	2.5	83.33	1.2 ± 0.13^e^	2.52 ± 0.17^a^
	5	66.66	1 ± 0.00^e^	1.95 ± 0.03^b^
	7.5	66.66	1.25 ± 0.13^e^	1.57 ± 0.14^cd^
	10	50	1 ± 0^e^	0.96 ± 0.09^e^
2-iP	2.5	83.33	1.20 ± 0.13^e^	0.53 ± 0.02^f^
	5	83.33	1.25 ± 0.16^e^	0.46 ± 0.05^f^
	7.5	66.66	1.50 ± 0.18^e^	0.58 ± 0.05^f^
	10	66.66	1 ± 0.00^e^	0.60 ± 0.04^f^
BAP	2.5	83.33	2.4 ± 0.16^d^	0.87 ± 0.04^e^
	5	83.33	5.25 ± 0.31^c^	0.93 ± 0.08^e^
	7.5	75.00	8.6 ± 0.26^b^	1.37 ± 0.07^d^
	10	83.33	12.20 ± 0.64^a^	1.68 ± 0.06^c^
TDZ	2.5	50	0 ± 0^f^	0 ± 0^g^
	5	66.66	0 ± 0^f^	0 ± 0^g^
	7.5	66.66	0 ± 0^f^	0 ± 0^g^
	10	66.66	0 ± 0^f^	0 ± 0^g^

Data was recorded after 4 weeks of culture. Each value represents the mean ± standard error. Treatment mean values followed by different letters in their superscript are significantly different from each other (*p* = 0.05) according to Duncan’s multiple range test

### Induction of roots from shoots

Regenerated shoots of *Andrographis alata* were cultured on ½ and ¼ strength MS nutrient medium containing 1, 2, 5, and 10 μM NAA, IBA, and IAA, and the results are presented in Table 2. There were significant differences in percentage of root induction, number of roots per explant, and root length among the different auxins and concentrations tested (Table 2). Optimum rooting percentage (100%) and number of roots (19/shoot) were realized when shoots were cultured on ¼ strength nutrient medium supplemented with 1 μM IBA (Fig. 1c). The greatest root length (3.06 cm) was also observed on the same medium. On MS medium lacking auxins, rooting of shoots was not observed. Medium supplemented with NAA and IAA also induced rooting of shoots; however, percentage of response, number of roots per shoot, and root length were inferior when compared to IBA-supplemented medium (Table 2). Micropropagated plants were successfully transferred to pots containing mixture of soil and cocopeat in equal concentrations (Fig. 1d).

**Table 2:** Effect of auxins supplemented MS medium on induction of roots from the shoots of *Andrographis alata*

Growth regulator	Concentration (μM)	Percentage of response	Mean number of roots per shoot	Average root length (cm)
MS half strength medium + auxins				
Control	0	0	0 ± 0^e^	0 ± 0^e^
NAA	1	0	0 ± 0^e^	0 ± 0^e^
	2	0	0 ± 0^e^	0 ± 0^e^
	5	0	0 ± 0^e^	0 ± 0^e^
	10	0	0 ± 0^e^	0 ± 0^e^
IBA	1	100	1.4 ± 0.16^c^	2.10 ± 0.07^b^
	2	100	5.6 ± 0.16^b^	1.48 ± 0.03^c^
	5	100	12.20 ± 1.0^a^	2.18 ± 0.07^b^
	10	16.66	1 ± 0.66^cd^	0.30 ± 0.04^d^
IAA	1	100	2 ± 0^c^	2.19 ± 0.07^b^
	2	100	2 ± 0.21^c^	2.80 ± 0.09^a^
	5	0	0 ± 0^e^	0 ± 0^e^
	10	0	0 ± 0^e^	0 ± 0^e^
MS quarter strength medium + auxins				
Control	0.00	0	0 ± 0^g^	0 ± 0^h^
NAA	1	100	4 ± 0.21^ef^	2.34 ± 0.04^c^
	2	100	11.80 ± 0.64^b^	2.42 ± 0.10^c^
	5	66.66	11.50 ± 0.42^b^	1.25 ± 0.10^g^
	10	66.66	12.33 ± 0.42^b^	1.30 ± 0.16^fg^
IBA	1	100	19.0 ± 1.65^a^	3.06 ± 0.07^ab^
	2	100	11.60 ± 0.54^b^	2.96 ± 0.10^b^
	5	100	4.80 ± 0.13d^e^	3.36 ± 0.11^a^
	10	100	8.0 ± 0.63^c^	2.80 ± 0.13^b^
IAA	1	66.66	4.25 ± 0.16de	1.85 ± 0.10^de^
	2	100	6.20 ± ±0.48^cd^	2.10 ± 0.07^cd^
	5	100	11.40 ± 0.80^b^	1.20 ± 0.20^g^
	10	66.66	2.0 ± 0^f^	1.62 ± 0.13^ef^

Data was recorded after 4 weeks of culture. Each value represents the mean ± standard error. Treatment means followed by different letters in their superscript are significantly different from each other (*p* = 0.05) according to Duncan’s multiple range test

### Genetic fidelity of regenerated plants

**Fig. 2:**
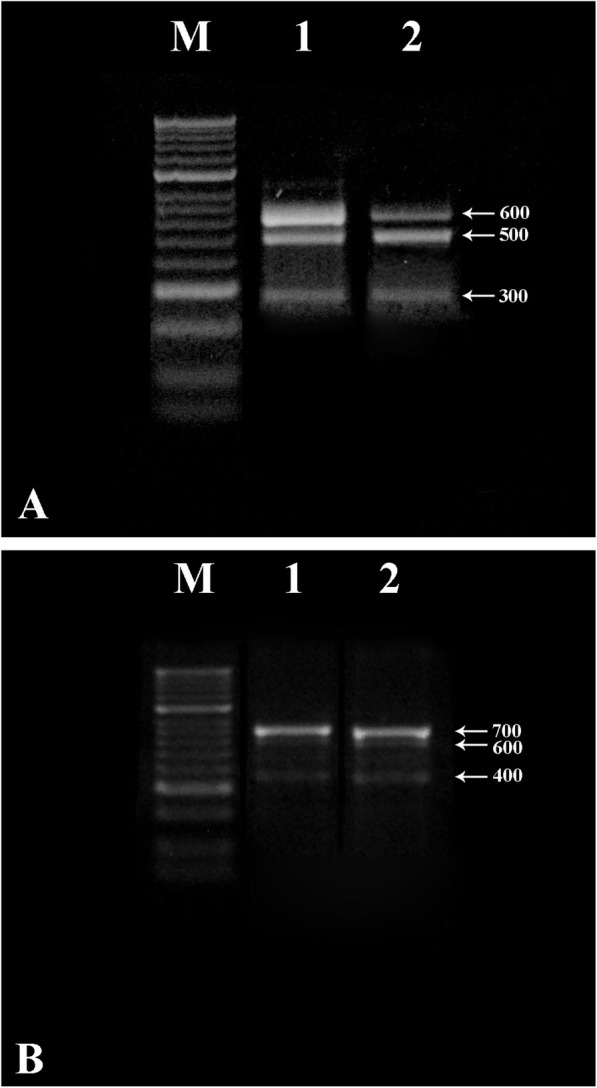
**a** RAPD profile of in vitro regenerated and mother plants of *A. alata* obtained with primer OPG-03. **b** ISSR profile of in vitro regenerated and mother plants of *A. alata* obtained from primer UBC-818 [lane M—DNA maker (1 kbp ladder); lane 1—in vitro regenerated plant; lane 2—mother plant of *A. alata* used as explants)

Genetic fidelity of in vitro regenerated *A. alata* was tested using RAPD and ISSR primers, and results obtained from two best RAPD (OPG-03) and ISSR (UBC-818) primers are presented in Fig. 2. Both the primers produced three monomorphic bands in mother plant as well as in vitro regenerated plants, which depict genetic fidelity of regenerated plants.

### Quantitative analysis of neoandrographolide

**Fig. 3:**
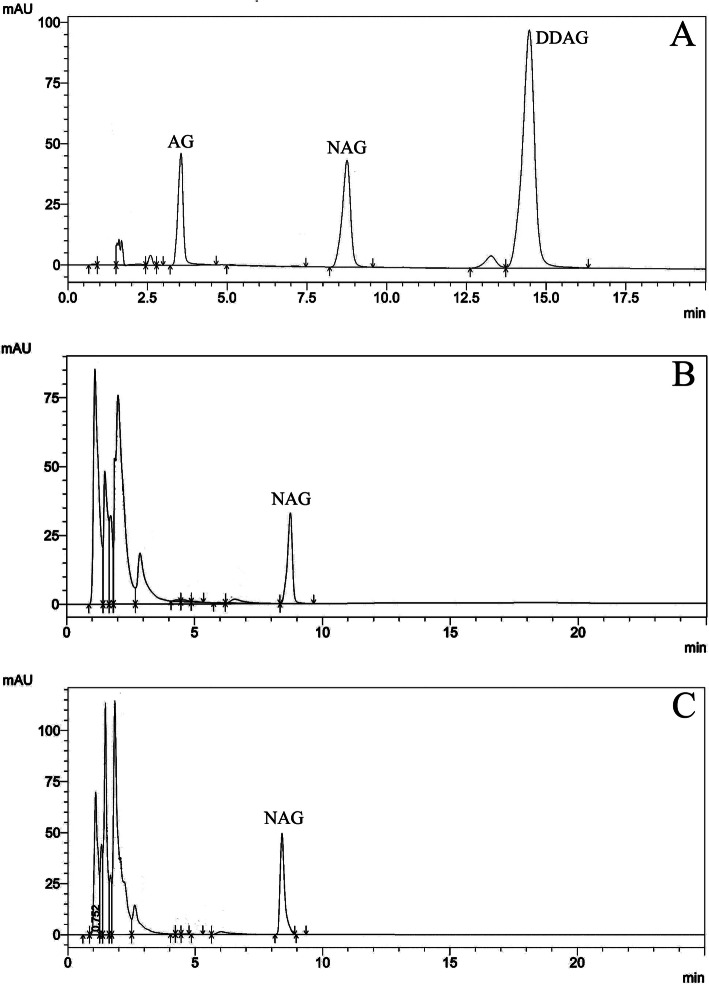
RP-HPLC elution profile of neoandrographolide (standard, panel **a**). RP-HPLC elution profile of neoandrographolide isolated from mother plant (panel **b**). RP-HPLC elution profile of neoandrographolide isolated from in vitro regenerated plants (panel **c**)

Amount of neoandrographolide was determined in parent and in vitro regenerated plants through RP-HPLC method. RP-HPLC chromatograms are presented in Fig. 3. Figure 3a represents peak areas of andrographolide (AG), neoandrographolide (NAG), and 14-deoxy-11,12-didehydroandrographolide (DDAG) standards. Figure 3 b and c depict the chromatograms of mother and in vitro regenerated plants respectively. Only neoandrographolide was detected in the mother and regenerated plants. Based on peak area measurements, the amount of neoandrographolide of mother and regenerated plants was found to be 32.32 mg/g DW.

## Discussion

The current study reveals that cytokinin-supplemented medium is appropriate for shoot regeneration from the nodal explants of *Andrographis alata* (Table 1). Among the varied cytokinins applied to the MS medium, BAP-containing medium was found suitable for regeneration of multiple shoots from nodal explants (Table 1). Multiple shoot regeneration from nodal explants on cytokinin medium was also reported in other medicinal plants such as *Artemisia nilagirica* var. *nilagirica* [17], *Artemisia japonica* [18], *Feronia limonia* [19], *Spilanthes oleracea* [20], and *Vitex trifolia* [21]. Among various cytokinins used, BAP was found excellent for axillary shoot induction (Table 1), and again of the several concentrations BAP tested, 10 μM BAP was superior in optimum axillary shoot induction (Table 1). These results are in congruent with earlier reports of BAP being used as a potent cytokinin for induction of multiple shoots in *Artemisia vulgaris* and *Puya berteroniana* [22–24]. Further, shoot regeneration frequency depends on type and concentration of cytokinin used (Table 1), such reports are already on record including *Andrographis paniculata* [25, 26], *Nothapodytes nimmoniana* [27], and *Enicostema axillare* [28].

Rooting of in vitro regenerated shoots is crucial for acclimatization of plants. Salt strength of medium and growth regulators supplemented to the medium is important factors which control the rooting of shoots. Auxins play a pivotal role in induction of adventitious root from in vitro regenerated shoots. Optimum root induction was realised on IBA-supplemented medium (1 μM; Fig. 1c; Table 2). Similar results were recorded in *Andrographis paniculata* [25], *Artemisia nilagirica* var. *nilagirica* [17], and *Artemisia japonica* [18] wherein IBA was best for induction of roots from regenerated shoots. It was reported that higher salt strength of medium inhibits rooting of in vitro regenerated shoots and invariably roots develop callus along with roots [17]. In the current study, optimal percentage of rooting, number of roots, and root length were recorded on the medium supplemented with ¼ strength MS nutrient medium with IBA (1 μM; Table 2). Similarly, reduced salt concentration of the culture medium was reported to be beneficial for root regeneration from shoots of *Feronia limonia* [21] and *Cattleya* [29].

Plant regenerated from axillary and apical buds are reported to possess genetic fidelity, whereas callus-mediated regeneration may depict tendency for genetic variation [30]. In the present study, genetic fidelity of regenerated plants was assessed using molecular markers. RAPD and ISSR analysis results demonstrated the development of monomorphic bands with the mother and in vitro regenerated plants which depicts genetic fidelity (Fig. 2). Such strategy adopting molecular technique for assessing genetic fidelity has been well documented in the literature [17, 20, 26].

Shoot cultures have been efficiently used for the production of secondary metabolites in vitro, for example, production of asiaticoside and madecassoside was demonstrated in shoot cultures of *Centella asiatica* [31]. Hypericins were produced from regenerated shoots of hairy St John’s-wort and spotted St. John’s-wort by Coste et al. [32]. In the current study, we have quantified the neoandrographolide in the regenerated plants, and results showed that the amount of neoandrographolide in in vitro regenerated plants was similar to mother plants (32.21 mg/g DW; Fig. 3).

## Conclusion

A simple, useful in vitro regeneration protocol was presented here for *Andrographis alata*. Highest frequency of shoots (12 shoots/explant) was regenerated on MS nutrient medium containing 10 μM BAP. Root regeneration from shoots was attained on ¼ strength MS medium containing 1 μM IBA. Molecular analysis showed the genetic fidelity of regenerated plants. Further, RP-HPLC analysis demonstrated that regenerated plants possessed optimum concentration of neoandrographolide. These results suggest that micropropagated plants of *Andrographis alata* are useful for both extraction of neoandrographolide for pharmaceutical use as well as for restoration of plants in nature.

## Data Availability

Not applicable

## Abbreviations

BAP: 6-Benzyl amino purine
DW: Dry weight
IAA: Indole acetic acid
IBA: Indole-butyric acid
KN: Kinetin
2-iP: 2-Isopentyl adenine
MS: Murashige and Skoog medium
NAA: 1-Napthlene acetic acid
RP-HPLC: Reverse phase high performance liquid chromatography
SARS-CoV-2: Severe acute respiratory syndrome corona virus 2
TDZ: Thidiazuron
